# DEMENTIA AND HEARING LOSS: A DOUBLE HIT ON PATIENT-PROVIDER COMMUNICATION?

**DOI:** 10.1093/geroni/igac059.607

**Published:** 2022-12-20

**Authors:** Danielle Powell, Nicholas Reed

**Affiliations:** Johns Hopkins University, Baltimore, Maryland, United States; Johns Hopkins Bloomberg School of Public Health, Baltimore, Maryland, United States

## Abstract

Hearing difficulty may adversely impact patient-provider communication and be exacerbated by the presence of other conditions like dementia. We examined the association between reported Alzheimer’s disease/dementia and reported difficulty communicating with providers. Using the 2019 Medicare Current Beneficiary Survey, we included participants were aged ≥65 years who reported functional difficulty hearing. Exposure was the presence of reported Alzheimer’s disease/dementia. Our outcome is reported difficulty communicating with medical providers. Multivariate logistic regression was used for association between the added presence of dementia and reported difficulty communicating with healthcare providers. Among 5,535 beneficiaries reporting hearing difficulty, diagnosis of Alzheimer’s disease or another dementia showed 2.76(95%CI:1.97-3.87) times greater odds of reporting difficulty communicating with providers compared to not reporting dementia. In summary, older adults with reported hearing difficulty and dementia may have increased difficulty communicating with medical providers. Findings suggest hearing management may aid in improving health outcomes for adults with dementia.

